# A carbohydrate approach for the formal total synthesis of (−)-aspergillide C

**DOI:** 10.3762/bjoc.10.329

**Published:** 2014-12-23

**Authors:** Pabbaraja Srihari, Namballa Hari Krishna, Ydhyam Sridhar, Ahmed Kamal

**Affiliations:** 1 Division of Natural Products Chemistry, CSIR-Indian Institute of Chemical Technology, Hyderabad, 500007, India; 2 Department of Medicinal Chemistry, National Institute of Pharmaceutical Education and Research, Hyderabad, 500037, India; 3 Medicinal Chemistry & Pharmacology, CSIR-Indian Institute of Chemical Technology, Hyderabad, 500007, India

**Keywords:** alkynylation, chiron approach, Ferrier-type *C*-glycosylation, macrolide

## Abstract

An enantioselective formal total synthesis of aspergillide C is accomplished using commercially available tri-*O*-acetyl-D-galactal employing a Ferrier-type *C*-glycosylation, utilizing a Trost hydrosilylation and protodesilylation as key reactions.

## Introduction

**Figure 1:**
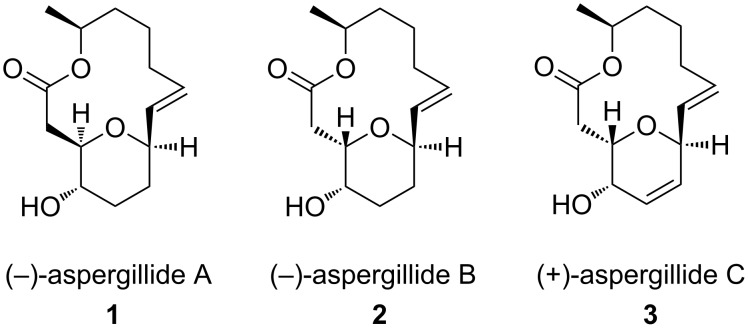
Structures of aspergillides.

Aspergillides A, B and C (Figure 1) (three, novel, bicyclic, 14-membered macrolides with 2,6-*cis* or *trans-*fused di- or tetrahydropyan rings) are unexpected, novel, secondary metabolites, isolated from the marine-derived fungus *Aspergillus ostianus* strain 01F313 in bromine-modified 1/2PD culture medium [1–4]. Interestingly, these compounds show cytotoxicity against mouse lymphocytic leukemia cells (L1210) with LD_50_ values of 2.1, 71.0, and 2.0 μg/mL, respectively [5–6]. The striking structural architecture and interesting biological properties have attracted significant attention from synthetic chemists with respect to their total synthesis [7–26] and have inspired medicinal chemists to synthesize diverse analogues in search for better potential molecules [27]. We have recently developed the total synthesis of these 14-membered macrolides and have accomplished the total syntheses of aspergillide B [15] and both enantiomers of aspergillide C [21]. The chiron approach has been a conventional strategy to achieve the total synthesis of complex, natural products with known handedness. Herein we disclose our strategy for the formal total synthesis of (−)-aspergillide C in a concise manner following the chiron approach.

## Retrosynthetic Analysis

**Scheme 1:**
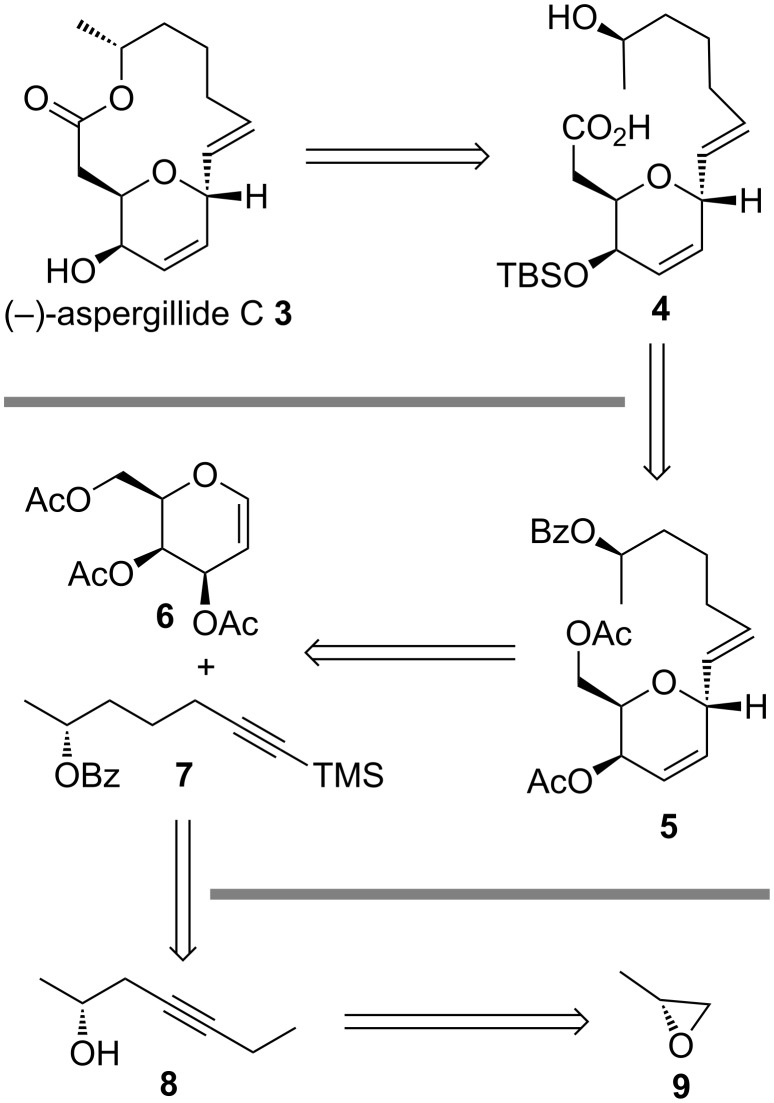
Retrosynthetic analysis for (−)-aspergillide C.

Through a retrosynthetic analysis, we envisaged that the macrolide **3** could be prepared from the *seco* acid **4** which can be easily accessed from **5** in five steps (Scheme 1). Compound **5**, in turn, can be synthesized from commercially available tri-*O*-acetyl-D-galactal (**6**) and alkyne **7** through a Ferrier-type *C*-glycosylation reaction followed by the reduction of the triple bond to the *trans* double bond. Alkyne **7** can be synthesized from alkyne **8** involving an isomerization reaction. Alkyne **8** was easily accessible from (*R*)-propylene oxide (**9**) through an epoxide opening reaction with 1-butyne.

## Results and Discussion

**Scheme 2:**
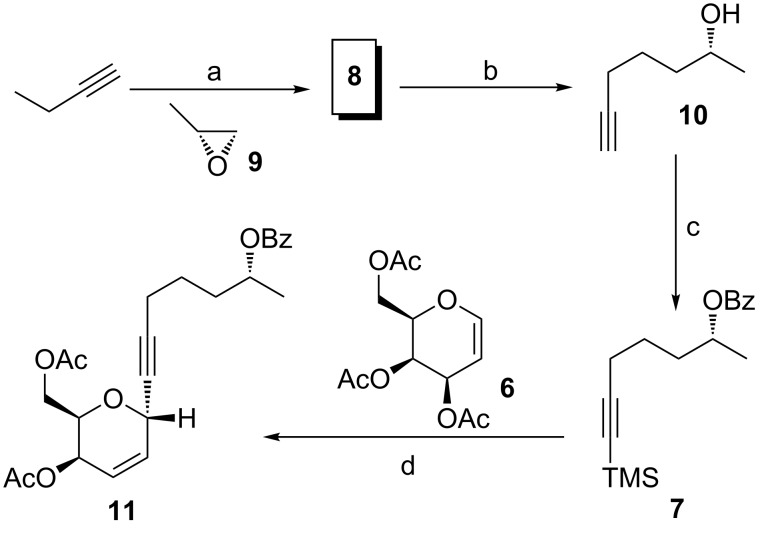
Synthesis of **11**. Reaction conditions: (a) *n*-BuLi, THF/HMPA (5:1), −78 °C to rt, 12 h, 95%; (b) Li, *t*-BuOK, H_2_N(CH_2_)_3_NH_2_, rt, 4 h, 92%; (c) i. *n*-BuLi, TMSCl, THF, −78 °C, 92%. ii. pyridine, C_6_H_5_COCl, CH_2_Cl_2_, 0 °C to rt, 1 h, 98%; (d) SnCl_4_, CH_2_Cl_2_, 0 °C to rt, 1 h, 85%.

**Figure 2:**
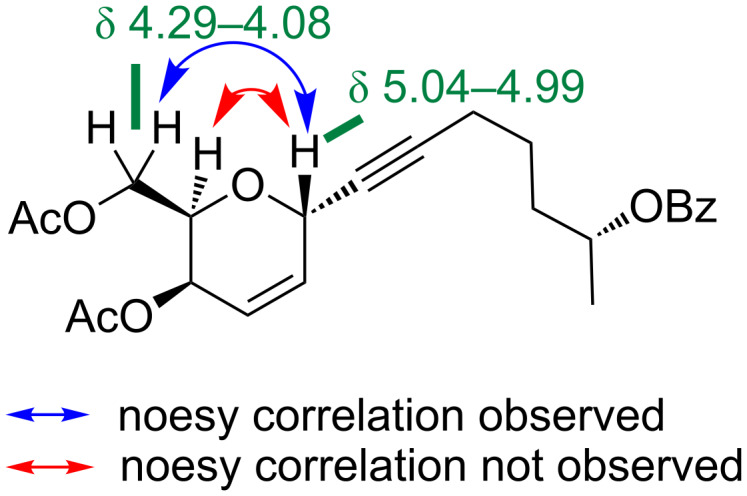
Key NOESY correlations observed in compound **11**.

In recent work on aspergillides, Achmatowicz adducts were utilized as the key source for the construction of the dihydropyran moiety and the side arm was synthesized using a Zipper rearrangement as a key reaction after an epoxide ring opening reaction of (*R*)-propylene oxide/(*S*)-propylene oxide with *n*-BuLi. In the present context, we have focused on a chiron approach for the synthesis of aspergillide C and thus chose commercially available tri-O-acetyl-D-galactal as the raw material, which can be directly utilized for the synthesis of the dihydropyran motif. The side arm fragment **10** was readily available in large amounts starting from (*R*)-propylene oxide (**9**) as shown in Scheme 2. We proceeded further by utilizing it for masking the functionalities. Compound **10** was disilylated using TMSCl in the presence of *n*-BuLi and later treated with 1 N HCl to get the free secondary alcohol which was further treated with benzoyl chloride in the presence of pyridine to produce the corresponding benzoyl ester **7** as a side chain fragment to be utilized for a Ferrier-type *C*-glycosylation reaction with the sugar tri-*O*-acetyl-D-galactal (**6**). Compound **6** was treated with silylated alkyne **7** in the presence of SnCl_4_ to afford alkynylated dihydropyran **11** as the only isomer, resulting in an 85% yield. The resulting dihydropyran ring was found to have a 2,6-*trans* configuration, owing to the attack of the alkyne from the opposite face of the acetyl-protected carbinol present in C6 position [28–31]. The geometry of the compound was also characterized by 2D NMR studies wherein significant NOESY correlations were observed for the C2 proton and the methylene protons of the carbinol moiety present on C6. Also, the NOESY correlation was absent between the C2 proton and the C6 proton (Figure 2).

**Scheme 3:**
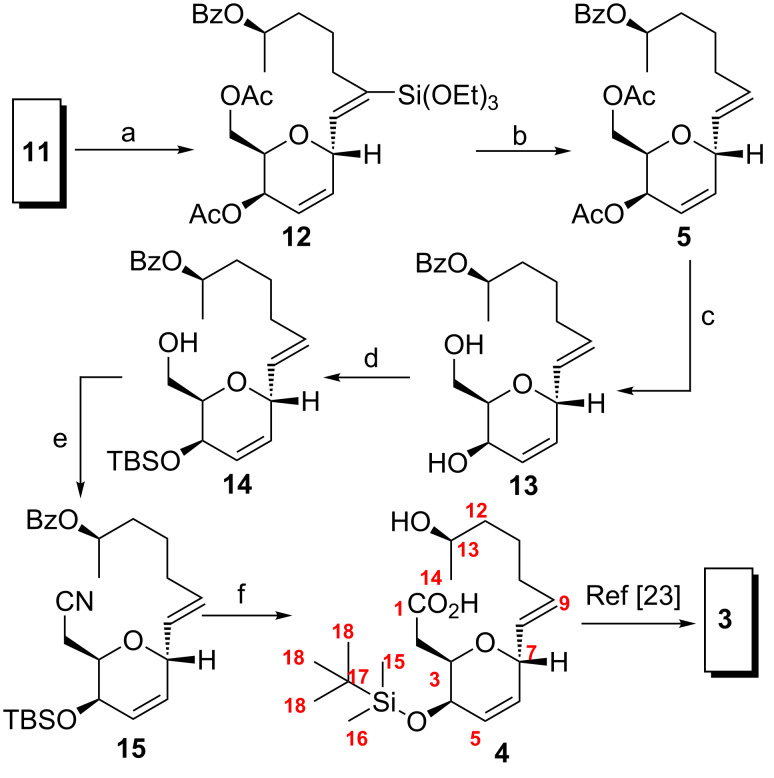
Synthesis of **4** and formal total synthesis of (−)-aspergillide C (**3**). Reaction conditions: (a) [Cp*(MeCN)_3_Ru]PF_6_, (EtO)_3_SiH, CH_2_Cl_2_, 0 °C to rt, 1 h, 95%; (b) HF/pyridine, pyridine, THF, 0 °C, 5 min, 90%; (c) cat. AcCl, MeOH, rt, 12 h, 90%; (d) (i) TBSCl, imidazole, DMAP, CH_2_Cl_2_, rt, 12 h, 95%; (ii) 70% HF in pyridine, THF, 0 °C, 12 h, 90%; (e) (i) Tf_2_O, 2,6-lutidine, −78 °C, 10 min; (ii) NaCN, DMF/DMSO, 18-crown-6, 90 °C, 30 min 88% overall for two steps; (f) 8 N NaOH, EtOH, 90 °C, 3 h, 75%.

After the alkynylation reaction, the stage was set to proceed further for the partial reduction of the alkyne moiety to obtain the *trans*-configured olefin. Based on our previous experience with this transformation, we proceeded directly with Trost’s hydrosilylation–protodesilylation protocol [32], where alkyne **11** was treated with excess triethoxysilane in the presence of a catalytic amount of [Cp*Ru(MeCN)_3_]PF_6_ to afford vinyl triethoxysilane **12** (Scheme 3). Compound **12** was then protodesilylated upon exposure to HF/pyridine to yield the desired *trans*-olefin **5**. Compound **5** possessed the required stereochemical configuration including the desired *trans*-olefin geometry, but required a one-carbon homologation to access the matched seco acid **4**. To achieve the one-carbon homologation with appropriate functionality, it was necessary to perform sequential chemoselective transformations. In this regard, we proceeded by selectively deprotecting the acetyl groups (without affecting the benzoate functionality) by employing acetyl chloride in anhydrous methanol to afford diol **13** [33]. A two-fold silylation and selective mono desilylation afforded primary alcohol **14** which was converted to its corresponding nitrile via the triflate. The nitrile functionality was hydrolysed with 8 N NaOH in ethanol to yield the *seco* acid **4**. The seco acid **4** was already utilized for the total synthesis of (−)-aspergillide C through macrolactonization and TBS deprotection as reported earlier by Kuwahara (Table 1) [23]. Thus, by synthesizing **4**, we have achieved the formal total synthesis of (−)-aspergillide C.

**Table 1:** Comparision of ^1^H and ^13^C NMR data of seco acid **4** with earlier data.

Position	Kuwahara data [23]		Our data	
	δ mult [*J* (Hz)] CDCl_3_, 500 MHz	^13^C NMR CDCl_3_,125 MHz	δ mult [*J* (Hz)] CDCl_3_, 300 MHz	^13^C NMR CDCl_3_, 75 MHz

1		174.9 (COOH)		175.0
2	2.72 (dd, *J* = 16.1, 8.8, 1H) 2.53 (dd, *J* = 16.1, 3.9, 1H)	37.7 (CH_2_)	2.71 (dd, *J* = 15.9, 9.1) 2.53 dd (15.9, 3.8)	37.8
3	3.93–3.96 (m, 1H)	68.1 (CH)	3.93–3.98 (m)	68.1
4	4.18 (dt, *J* = 8.8, 3.2, 1H)	69.5 (CH)	4.18 (dt *J* = 9.0, 3.2)	69.6
5	5.85–5.91 (m, 2H)	126.7 (CH)	5.84–5.91 (m)	126.7
6		127.5 (CH)		127.5
7	4.69 (d, *J* = 5.9, 1H)	72.5 (CH)	4.68 (d, *J* = 5.3)	72.4
8	5.51 (dd, *J* = 15.6, 5.9, 1H)	130.7 (CH)	5.51 (dd, *J* = 15.8, 6.0)	130.7
9	5.66 (dt, *J* = 15.6, 6.6, 1H)	134.8 (CH)	5.77–5.60 (m)	134.6
10	2.00–2.08 (m, 1H) 2.08–2.16 (m, 1H)	36.0 (CH_2_)	1.96–2.20 (m)	35.9
11	1.34–1.52 (m, 3H) 1.55–1.63 (m, 1H)	24.4 (CH_2_)	1.34–1.52 (m)	24.5
12		31.7 (CH_2_)		31.7
13	3.82 (sex, *J* = 6.3, 1H)	64.4 (CH)	3.82 (sex, *J* = 6.0)	64.4
14	1.18 (d, *J* = 6.3, 3H)	22.8 (CH_3_)	1.18 (d *J* = 6.0)	22.9
15	0.093 (s, 3H)	−4.6 (Si-CH_3_)	0.09 (s)	−4.6
16	0.086 (s, 3H)	−4.1 (Si-CH_3_)	0.08–0.10 (m)	−4.1
17	–	18.2 (C)	–	18.2
18	0.90 (s, 9H)	25.9 (C(CH_3_)_3_)	0.90 (s)	25.9
OH	1.25 (s, 1H)	–	1.25 (s)	–

## Conclusion

In conclusion, a formal total synthesis of (−)-aspergillide C has been achieved through a concise, stereocontrolled synthesis of the known key intermediate **4** in 8 steps with an overall yield of 36.9% starting from commercially available tri-*O*-acetyl-D-galactal. A *C*-glycosidation, Trost’s hydrosilylation and protodesilylation protocol have been used as the key steps for achieving the formal total synthesis.

## Supporting Information

File 1 Experimental details and analytical data.
