# Supplementary material for: A carbohydrate approach for the formal total synthesis of (−)-aspergillide C
Source: Beilstein J Org Chem. 2014 Dec 23;10:3122–6. doi: 10.3762/bjoc.10.329 (PMC4311580; doi:10.3762/bjoc.10.329)
Supplement: File 1 — Experimental details and analytical data. [file Beilstein_J_Org_Chem-10-3122-s001.pdf]

Supporting Information

for

A carbohydrate approach for the formal total synthesis of (−)-aspergillide C

Pabbaraja Srihari*[1], Namballa Hari Krishna[1,2,3], Ydhyam Sridhar[1] and Ahmed Kamal[2,3]

Address: [1]Division of Natural Products Chemistry, CSIR-Indian Institute of Chemical Technology, Hyderabad, 500007, India, [2]Department of Medicinal Chemistry, National Institute of Pharmaceutical Education and Research, Hyderabad, 500037, India and [3]Medicinal Chemistry & Pharmacology, CSIR-Indian Institute of Chemical Technology, Hyderabad, 500007, India

Email: Pabbaraja Srihari - srihari@iict.res.in

* Corresponding author

Experimental details and analytical data

Experimental Page S2–S10

Spectra Page S11–S19

Experimental

General Methods.^1H NMR and ^{13}C NMR spectra were recorded in $CDCl_3$ as solvent on 300 MHz or 500 MHz spectrometer at ambient temperature. The chemical shifts are reported in ppm downfield from TMS as internal standard and signal patterns are indicated as follows: s = singlet, d = doublet, t = triplet, q = quartet, sext = sextet, m = multiplet, br = broad. The coupling constants *J* are given in Hz. FTIR spectra were recorded on KBr pellets and reported in wave number (cm^{-1}). Optical rotations were measured on digital polarimeter using a 1 mL cell with 1 dm path length. For low (MS) and High (HRMS) resolution, *m*/*z* ratios are reported as values in atomic mass units. Mass analysis was done in ESI mode. All reagents were reagent grade and used without further purification unless specified otherwise. Solvents for reactions were distilled prior to use: THF, and diethyl ether were distilled from Na and benzophenone ketyl; CH_2Cl_2 from CaH_2. All air- or moisture-sensitive reactions were conducted under a nitrogen or argon atmosphere in flame-dried or oven-dried glassware with magnetic stirring. Column chromatography was carried out using silica gel (100–200 mesh) packed in glass columns. Technical grade ethyl acetate and petroleum ether used for column chromatography were distilled prior to use.

(*R*)-7-(Trimethylsilyl)hept-6-yn-2-yl benzoate (7). To a magnetically stirred solution of mono-silylated alcohol **10** (6.7 g, 36.4 mmol) in CH_2Cl_2 (60 mL), pyridine (5.9 mL, 72.8 mmol) was added at 0 °C followed by C_6H_5COCl (5.5 mL, 54.6 mmol) at the same temperature. The alcohol **10** was consumed within 2 h, and the reaction was then quenched with 1 N HCl (110 mL, 110

mmol) at 0 °C and extracted with CH_2Cl_2 (3 x 20 mL). The organic layer was washed with water (30 mL) and sat. aq. $NaHCO_3$ (20 mL), and dried with anhydrous Na_2SO_4. Evaporation of CH_2Cl_2 *in vacuo* gave the crude product, which was purified by column chromatography (hexane/EtOAc 9.7:0.3) to afford pure compound **7** as a pale yellow oil (10.3 g, 98%); *Rf* = 0.5 (hexane/EtOAc 9.5:0.5); ^{1}H NMR (300 MHz, $CDCl_3$): δ 8.04 (d, *J* = 6.8 Hz, 2H), 7.60-7.52 (m, 1H), 7.48-7.40 (m, 2H), 5.31-5.12 (m, 1H), 2.27 (t, *J* = 6.8 Hz, 2H), 2.00-1.60 (m, 4H), 1.36 (dd, *J* = 6.8, 2.3 Hz, 3H), 0.15 (s, 9H). ppm; ^{13}C NMR (75 MHz, $CDCl_3$): δ 166.1, 132.7, 130.7, 129.5, 128.3, 106.8, 84.9, 71.1, 35.1, 24.5, 20.1, 19.7, 0.1 ppm.

(*R*)-7-((2*S*,5*R*,6*R*)-5-Acetoxy-6-(acetoxymethyl)-5,6-dihydro-2*H*-pyran-2-yl)hept-6-yn-2-yl benzoate (11). To a magnetically stirred solution of tri-O-acetyl-D-galactal (2.24 g, 8.24 mmol) and **7** (2.49 g, 8.65 mmol) in CH_2Cl_2 (40 mL), $SnCl_4$ (1 M in heptane, 4.2 mL, 0.52 mmol) was added at 0 °C. The ice bath was then removed and stirring was continued at room temp. Within 1 h, all the starting materials were consumed (reaction monitored by TLC) and the reaction was quenched with sat. aq. $NaHCO_3$ (20 mL) at 0 °C, diluted with water (20 mL), stirred at room temp. for 1 h, then extracted with CH_2Cl_2 (3 x 10 mL). The organic layer was washed with brine (20 mL), and dried with anhydrous Na_2SO_4. Evaporation of CH_2Cl_2 *in vacuo* gave the crude product, which was subjected to purification on silica gel column chromatography (hexane/EtOAc, 9:1) to give the desired product **11** as light-yellow oil (3.57 g, 85%). R_f = 0.5 (hexane/EtOAc, 7:3); $[\alpha]_D^{24}$ = -47 (*c* 0.3, $CHCl_3$); IR (KBr): 2939, 2216, 1744, 1715, 1451, 1373, 1276, 1230, 715 cm^{-1}; ^{1}H NMR (300

MHz, $CDCl_3$): δ 8.08-8.00 (m, 2H), 7.60-7.52 (m, 1H), 7.48-7.39 (m, 2H), 6.04 (dd, J = 10.0, 6.4 Hz, 1H), 5.96 (ddd, J = 10.0, 5.1, 1.5 Hz, 1H), 5.25-5.13 (m, 1H), 5.05 (dd, J = 5.1, 2.3 Hz, 1H), 5.04-4.99 (m, 1H), 4.33 (ddd, J = 7.4, 5.3, 2.5 Hz, 1H), 4.29-4.08 (m, 2H), 2.29 (td, J = 8.9, 7.0, 1.9 Hz, 2H), 2.08 (s, 3H), 2.06 (s, 3H), 1.90-1.73 (m, 2H), 1.73-1.55 (m, 2H), 1.36 (d, J = 6.2 Hz, 3H) ppm; ^{13}C NMR (75 MHz, $CDCl_3$): δ 170.3, 170.4, 160.1, 132.8, 132.6, 130.6, 129.4, 128.3, 121.8, 87.2, 75.8, 70.9, 69.3, 64.0, 63.3, 62.8, 35.0, 24.3, 20.8, 20.7, 20.0, 18.6 ppm; HRMS calcd for $C_{24}H_{28}O_7Na$ $[M + Na]^+$ 451.1727, found 451.1724.

(*R*,*E*)-7-((2*R*,5*R*,6*R*)-5-Acetoxy-6-(acetoxymethyl)-5,6-dihydro-2*H*-pyran-2-yl)hept-6-en-6-triethoxysilyl-2-yl benzoate (12). To a vigorously stirring solution of alkyne **11** (3.0 g, 5.88 mmol) and $(EtO)_3SiH$ (4.42 mL, 23.53 mmol) in CH_2Cl_2 (60 mL), $[Cp^*(MeCN)_3Ru]PF_6$ (30 mg, 0.059 mmol) was added at 0 °C under an argon atmosphere, and the ice bath was then removed. Within 1 h, alkyne **11** was completely consumed (reaction monitored by TLC). Evaporation of CH_2Cl_2 in *vacuo* gave the crude vinyl siloxane**12**, which was subjected to silica gel column chromatography (hexane/EtOAc, 9:1) to give the desired product **12** (3.95 g, 95%) as light yellow oil. R_f = 0.45 (hexane/EtOAc, 8:2); ^1H NMR (300 MHz, $CDCl_3$): δ 8.04-8.00 (m, 2H), 7.55-7.51 (m, 1H), 7.44-7.39 (m, 2H), 6.19-6.16 (m, 1H), 5.98 (dd, J = 10.2, 3.2 Hz, 1H), 5.92 (ddd, J = 10.2, 5.0, 2.0 Hz, 1H), 5.25-5.21 (m, 1H), 5.19-5.13 (m, 1H), 5.06 (dd, J = 4.9, 2.6 Hz, 1H), 4.20 (dd, J = 5.0, 2.8 Hz, 1H), 4.19-4.16 (m, 2H), 3.79 (dq, J = 14.0, 7.0, 0.9 Hz, 6H), 2.19-2.13 (m, 2H), 2.07 (s, 3H), 2.02 (s, 3H), 1.79-1.69 (m, 2H), 1.65-1.45 (m, 2H), 1.33 (d, J = 6.3 Hz, 3H)

1.19 (t, *J* = 7.0 Hz, 9H) ppm; ^{13}CNMR (75 MHz, $CDCl_3$): *δ* 170.7, 170.6, 166.1, 140.8, 136.4, 134.9, 132.7, 130.8, 129.4, 128.2, 121.1, 72.1, 71.4, 68.1, 63.5, 62.8, 58.4, 37.2, 35.7, 29.6, 20.9, 20.7, 20.0, 18.1 ppm.

(*R*,*E*)-7-((2*R*,5*R*,6*R*)-5-Acetoxy-6-(acetoxymethyl)-5,6-dihydro-2*H*-pyran-2-yl)hept-6-en-2-yl benzoate (5). To a vigorously stirring solution of vinyl siloxane **12** (3.0 g, 5.06 mmol) in THF (40 mL), pyridine (4.8 mL, 58.8 mmol) followed by HF/pyridine (70:30 solution, 0.82 mL, 29.4 mmol) were added dropwise at 0 °C. After all vinyl siloxane was consumed (~5 min; reaction monitored by TLC), the reaction was quenched with water (20 mL), neutralized with 0.5 N HCl (35 mL), and extracted with EtOAc (3 x 30 mL). The organic layers were washed with water (60 mL) and brine (40 mL), and dried with anhydrous Na_2SO_4. Evaporation of solvent *in vacuo* gave the crude product, which was subjected to silica gel column chromatography (hexane/EtOAc, 8.5:1.5) to give the desired product **5** as a light-yellow oil (1.96 g, 90%). R_f = 0.5 (hexane/EtOAc, 3:2); $[\alpha]_D^{24}$ = -14 (*c* 0.3, $CHCl_3$); IR (KBr): 2937, 1745, 1715, 1624, 1451, 1371, 1276, 1229, 1113, 716 cm^{-1}; ^{1}H NMR (300 MHz, $CDCl_3$): *δ* 8.07-8.00 (m, 2H), 7.60-7.52 (m, 1H), 7.48-7.39 (m, 2H), 6.11-5.97 (m, 2H), 5.75-5.60 (m, 1H), 5.51 (dd, *J* = 15.8, 5.1 Hz, 1H), 5.24-5.11 (m, 1H), 5.03 (dd, *J* = 4.7, 2.5 Hz, 1H), 4.81-4.74 (m, 1H), 4.27-4.10 (m, 2H), 4.06 (ddd, *J* = 8.7, 6.4, 2.5 Hz, 1H), 2.20-2.10 (m,2H), 2.09 (s, 3H), 2.03 (s, 3H), 1.83-1.40 (m, 4H), 1.35 (d, *J* = 6.4 Hz, 3H) ppm; ^{13}CNMR (75 MHz, $CDCl_3$): *δ* 170.6, 170.5, 166.1, 134.6, 133.9, 132.7, 130.7, 129.4, 128.2, 126.3, 122.3, 72.6, 71.3, 67.9, 63.8, 63.1, 35.4, 32.1, 24.8, 20.9, 20.7, 20.0 ppm; HRMS calcd for $C_{24}H_{30}O_7Na$ $[M + Na]^+$ 453.1884, found 453.1883.

(*R*,*E*)-7-((2*R*,5*R*,6*R*)-5-Hydroxy-6-(hydroxymethyl)-5,6-dihydro-2*H*-pyran-2-yl)hept-6-en-2-yl benzoate (13). To the magnetically stirring solution of **5** (1.1 g, 2.15 mmol) in dry methanol (20 mL), acetyl chloride (0.03 mL, 0.43 mmol) was added at 0 ^{o}C and stirring was continued while allowing the reaction mixture to attain room temperature. After complete consumption of starting material (ca. 4 h, as monitored by TLC) solvent was removed under reduced pressure to give the crude mass which was subjected to column chromatography (EtOAc/Hexane 3:7) to give the diol **13** as colorless oil. R_f = 0.5 (hexane/EtOAc, 3:7); $[\alpha]_D^{24}$ = -31 (*c* 0.25, $CHCl_3$); IR (KBr): 3407, 2934, 1713, 1452, 1380, 1278, 1229, 1112, 714 cm–1; ^{1}H NMR (500 MHz, $CDCl_3$): δ 8.08-7.99 (m, 2H), 7.60-7.51 (m, 1H), 7.49-7.39 (m, 2H), 6.05 (ddd, *J* = 10.0, 5.3, 1.7 Hz, 1H), 5.94 (dd, *J* = 10.2, 3.4 Hz, 1H), 5.72-5.48 (m, 2H), 5.23-5.09 (m, 1H), 4.75-4.68 (m, 1H), 3.94-3.72 (m, 4H), 2.66-2.41 (bs, 1H), 2.40-2.20 (bs, 1H), 2.20-2.02 (m, 2H), 1.83-1.40 (m, 4H), 1.34 (d, *J* = 6.2 Hz, 3H), ppm; ^{13}CNMR (75 MHz, $CDCl_3$): δ 166.2, 134.6, 132.8, 132.0, 130.7, 129.5, 128.3, 126.9, 126.6, 73.0, 71.6, 71.4, 63.0, 62.9, 35.4, 32.1, 24.8, 20.0 ppm; HRMS calcd for $C_{20}H_{26}O_5Na$ [M + Na]$^+$ 369.1673, found 369.1672.

(*R*,*E*)-7-((2*R*,5*R*,6*R*)-5-((*tert*-Butyldimethylsilyl)oxy)-6-(hydroxymethyl)-5,6-dihydro-2*H*-pyran-2-yl)hept-6-en-2-yl benzoate (14). To a magnetically stirring solution of compound **13** (1 g, 2.34 mmol) in CH_2Cl_2 (15 mL), imidazole (0.95 g, 1.4 mmol), TBSCl (1.06 g, 7 mmol), and DMAP (0.15 g, 1.2 mmol) were added at 0 °C under nitrogen atmosphere and stirring was continued until complete consumption of starting material was observed (ca. 12 h). The reaction mixture was quenched with sat. aq. NH_4Cl (10 mL), diluted

with water (10 mL), and extracted with CH_2Cl_2 (3 x 10 mL). The combined organic layer was washed with brine (10 mL) and dried over anhydrous Na_2SO_4. Evaporation of CH_2Cl_2 *in vacuo* gave the crude product, which was subjected to silica gel column chromatography to give the desired product **14a** as a colorless liquid.

To the magnetically stirring solution of **14a** (7.0 g, 16.8 mmol) in THF (90 mL) in a teflon vial, HF/pyridine (7:3 complex, 4.4 mL, 0.15 mmol) was added at 0 ^oC and stirring continued at the same temperature while monitoring the reaction progress by TLC analysis. After complete consumption of starting material (ca. 12 h) the reaction mixture was neutralized with solid $NaHCO_3$ and the filtrate was concentrated to give the crude mass, which upon column purification (EtOAc/Hexane 1.5:8.5) gave the mono TBS ether **14** as colorless oil (4.6 g, 90%).

14a:(1.30 g,98%); R_f = 0.5 (hexane/EtOAc, 9.5:0.5); $[\alpha]_D^{24}$ = -8 (*c* 0.25, $CHCl_3$); IR (KBr): 2954, 2930, 2857, 1718, 1636, 1466, 1360, 1275, 1254, 1112, 836, 775 cm–1; ^1H NMR (500 MHz, $CDCl_3$): δ 8.10-8.04 (m, 2H), 7.62-7.54 (m, 1H), 7.51-7.42 (m, 2H), 5.96-5.82 (m, 2H), 5.76-5.62 (m, 1H), 5.61 (dd, *J* = 15.7, 5.3 Hz, 1H), 5.26-5.12 (m, 1H), 4.74-4.68 (m, 1H), 4.05-3.99 (m, 1H), 3.89-3.68 (m, 2H), 3.29-3.23 (m, 1H), 2.21-2.06 (m, 2H), 1.85-1.41 (m, 4H), 1.36 (d, *J* = 6.2 Hz, 3H), 0.92 (s, 9H), 0.91 (s, 9H), 0.11 (s, 6H), 0.08 (s, 6H) ppm; ^{13}CNMR (75 MHz, $CDCl_3$): δ 166.1, 133.0, 132.7, 131.0, 130.8, 129.5, 128.2, 127.6, 126.9, 73.6, 72.2, 71.4, 63.0, 62.5, 35.6, 32.2, 25.9, 25.7, 24.9, 20.1, 18.3, 18.2, -4.1, -4.6, -5.2, -5.3 ppm; HRMS calcd for $C_{32}H_{54}O_5NaSi_2$ $[M + Na]^+$ 597.3402, found 597.3396.

14: R_f = 0.5 (hexane/EtOAc, 7:3); $[\alpha]_D^{24}$ = -9 (*c* 0.25, $CHCl_3$); IR (KBr): 3496, 2932, 2858, 1715, 1625, 1454, 1356, 1277, 1113, 838, 777 cm^{-1}; ^1H NMR (300 MHz, $CDCl_3$): δ 8.05-8.00 (m, 2H), 7.58-7.50 (m, 1H), 7.47-7.38 (m, 2H), 5.89-5.78 (m, 2H), 5.69-5.56 (m, 1H), 5.48 (dd, *J* = 15.9, 5.3 Hz, 1H), 5.21-5.09 (m, 1H), 4.71-4.64 (m, 1H), 4.09-4.01 (m, 1H), 3.91-3.77 (m, 2H), 3.72 (dd, *J* = 9.8, 3.0 Hz, 1H), 2.43-2.21 (bs,1H), 2.14-2.01 (m, 2H), 1.81-1.38 (m, 4H), 1.32 (d, *J* = 6.0 Hz, 3H), 0.88 (s, 9H), 0.08 (s, 6H) ppm; ^{13}CNMR (75 MHz, $CDCl_3$): δ 166.2, 134.1, 132.7, 131.0, 130.8, 129.5, 128.3, 127.7, 126.6, 72.7, 72.0, 71.4, 64.3, 62.7, 35.5, 32.1, 25.8, 24.9, 20.0, 18.1, -4.2, -4.7 ppm; HRMS calcd for $C_{26}H_{40}O_5NaSi$ $[M + Na]^+$ 483.2537, found 483.2538.

(*R*,*E*)-7-((2*R*,5*R*,6*R*)-5-((*tert*-Butyldimethylsilyl)oxy)-6-(cyanomethyl)-5,6-dihydro-2*H*-pyran-2-yl)hept-6-en-2-yl benzoate (15). To the stirring solution of alcohol **14** (0.27 g, 0.6 mmol) in CH_2Cl_2 (5 mL), 2,6-lutidine (0.21 mL, 1.8 mmol) followed by Tf_2O (0.11 mL, 0.66 mmol) were added at 0 ^oC. Within 5 min entire starting material was consumed (reaction monitored by TLC) and the reaction mixture was quenched with sat. aq. NH_4Cl (5 mL) and extracted with CH_2Cl_2 (2 x 10 mL). The combined organic layers were dried over anhydrous Na_2SO_4, filtered, concentrated under reduced pressure to give the triflate. To the triflate dissolved in DMF (5 mL), 18-crown-6 (31 mg, 0.12 mmol) followed by NaCN (0.15 g, 3.0 mmol) were added and the mixture was heated to 90 ^oC. After the entire starting material was consumed (~30 min) the reaction mixture was diluted with ice cold water (20 mL) and extracted with Et_2O (3 x 10 mL), the combined extracts were washed with brine (10 mL), dried over anhydrous Na_2SO_4, filtered and concentrated under reduced

pressure to give crude product which upon column purification (Hexane/EtOAc 9/1) gave nitrile **15** (0.245 g, 88%) as colour less oil. R_f = 0.5 (hexane/EtOAc, 3:7); $[\alpha]_D^{24}$ = -122 (*c* 0.25, $CHCl_3$); IR (KBr): 2932, 2858, 2251, 1715, 1456, 1359, 1275, 1113, 837, 775 cm^{-1}; ^1H NMR (300 MHz, $CDCl_3$): δ 8.06-8.02 (m, 2H), 7.55 (tt, *J* = 1H), 7.46-7.42 (m, 2H), 5.88-5.82 (m, 2H), 5.68 (dddd, 1H), 5.48 (qt, *J* = 1H), 5.20-5.13 (m, 1H), 4.69-4.64 (m, 1H), 4.10-4.00 (m, 2H), 2.66-2.62 (m, 2H), 2.15-2.06 (m, 2H), 1.80-1.70 (m, 1H), 1.69-1.59 (m, 1H), 1.58-1.40 (m, 2H), 1.35 (d, *J* = 6.4 Hz, 3H), 0.90 (s, 9H), 0.12 (s, 3H), 0.10 (s, 3H) ppm; ^{13}CNMR (75 MHz, $CDCl_3$): δ 166.1, 134.5, 132.7, 130.7, 129.4, 128.2, 126.9, 126.1, 117.9, 72.1, 71.3, 69.2, 63.3, 35.4, 32.0, 25.7, 24.7, 20.0, 19.0, 18.1, -4.1, -4.7 ppm; HRMS calcd for $C_{27}H_{39}O_4NNaSi$ $[M + Na]^+$ 492.2541, found 492.2541.

2-((2*R*,3*R*,6*R*)-3-((*tert*-Butyldimethylsilyl)oxy)-6-((*R*,*E*)-6-hydroxyhept-1-en-1-yl)-3,6-dihydro-2*H*-pyran-2-yl)acetic acid (4). Aqueous 8 N NaOH solution was added to a solution of cyanide **15** (0.1 g, 0.21 mmol) in EtOH and refluxed for 3 h. The reaction was neutralized using 3 N HCl solution after cooling to rt. The mixture was diluted with water and extracted with ethyl acetate (3 × 20 mL). The combined organic layers were washed with brine, dried over anhydrous Na_2SO_4, concentrated in *vacuo*, and purified by column chromatography to give **4** (0.06 g, 75%) as a pale yellow oil. R_f = 0.3 (hexane/EtOAc, 1:1); $[\alpha]_D^{24}$ = -145.0 (c 0.27, $CHCl_3$); IR (KBr): 3422, 3037, 2999, 1699, 1050, 830cm^{-1}; ^1H NMR (300 MHz, $CDCl_3$): δ 5.91-5.84 (m, 2H), 5.77-5.60 (m, 1H), 5.51 (dd, *J* = 15.8, 6.0 Hz, 2H), 4.68 (d, *J* = 5.3 Hz, 1H), 4.18 (dt, *J* = 9.0, 3.2 Hz, 1H), 3.93-3.98 (m, 1H), 3.82 (sex, *J* = 6.0 Hz, 1H),

2.71 (dd, J = 15.9, 9.1 Hz, 1H), 2.53 (dd, J = 15.9, 3.8 Hz, 1H), 1.96-2.20 (m, 2H), 1.63-1.38 (m, 2H), 1.25 (s, 1H, OH), 1.18 (d, J = 6.0 Hz, 3H), 0.9 (s, 9H), 0.10-0.08 (m, 6H). ppm; ^{13}CNMR (75 MHz, $CDCl_3$): δ 175.0, 134.6, 130.7, 127.5, 126.7, 72.4, 69.6, 68.1, 64.4, 37.8, 35.9, 31.7, 25.9, 24.5, 22.9, 18.2, -4.1, -4.6 ppm. HRMS calcd for $C_{20}H_{37}O_5Si$ $[M + Na]^+$ 385.2366, found 385.2384.

^{1}H and ^{13}C NMR spectra of key compounds:

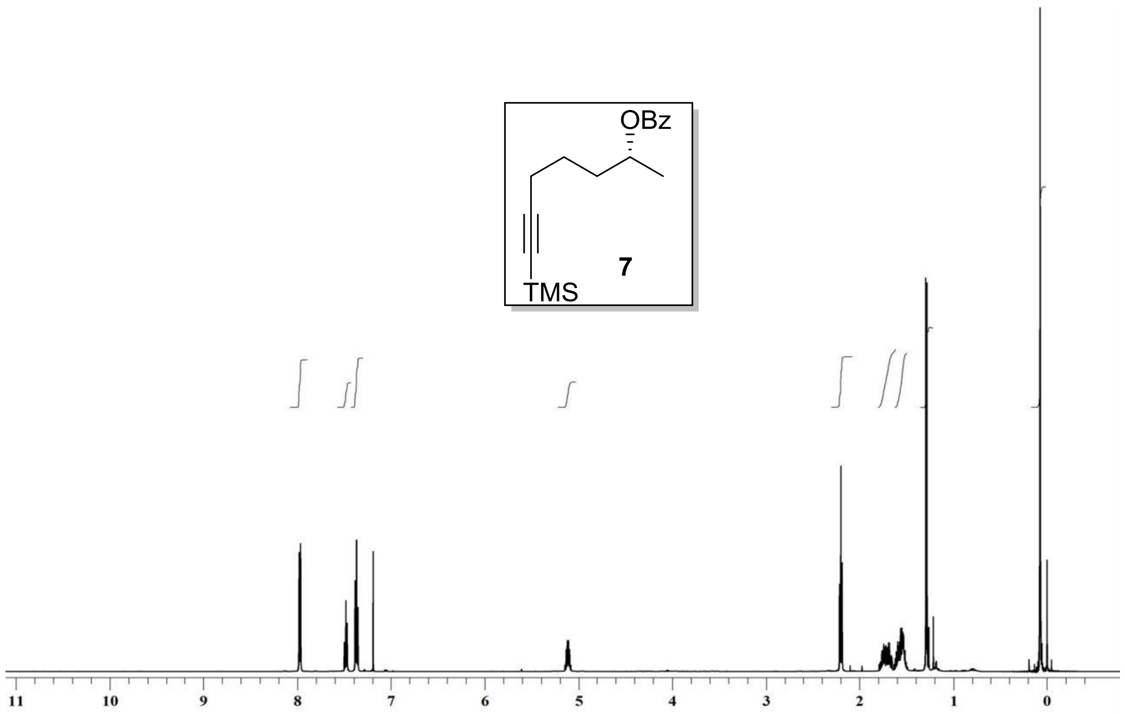

^{1}H NMR Spectrum of 7 (300 MHz, $CDCl_3$)

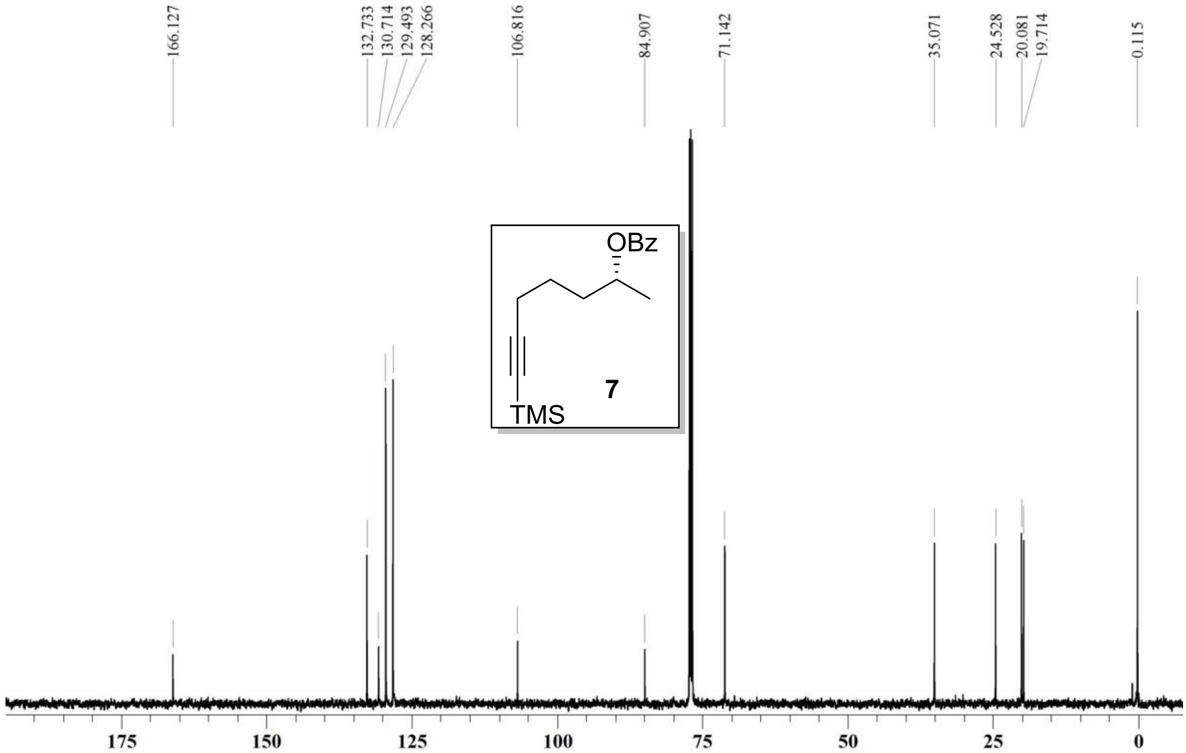

^{13}C NMR Spectrum of 7 (75 MHz, $CDCl_3$)

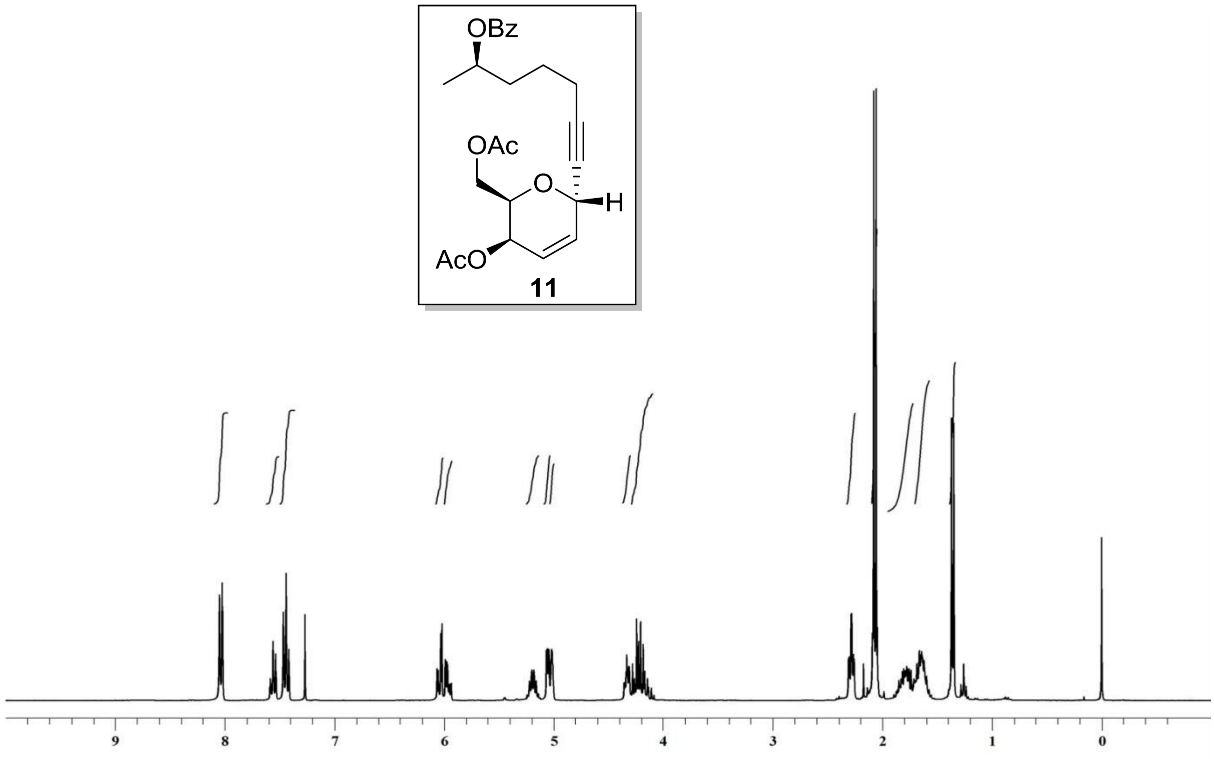

^{1}H NMR Spectrum of 11 (300 MHz, $CDCl_3$)

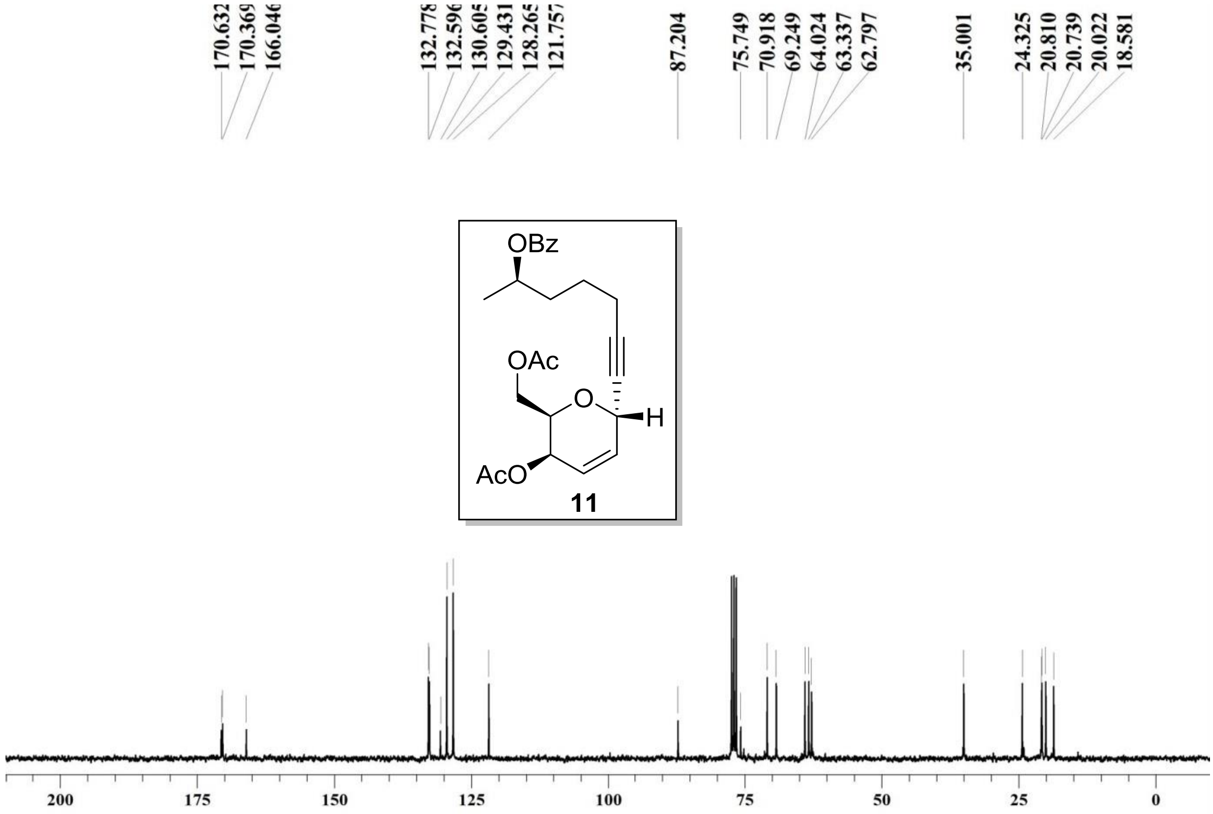

^{13}C NMR Spectrum of 11 (75 MHz, $CDCl_3$)

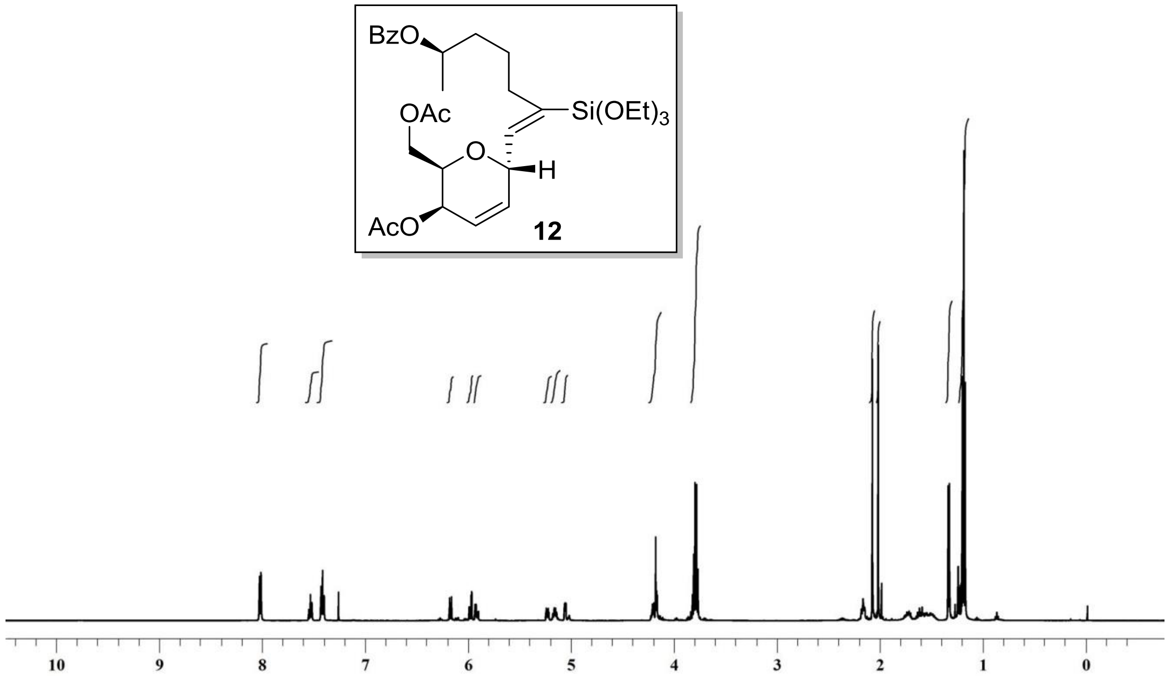

^{1}H NMR Spectrum of 12 (500 MHz, $CDCl_3$)

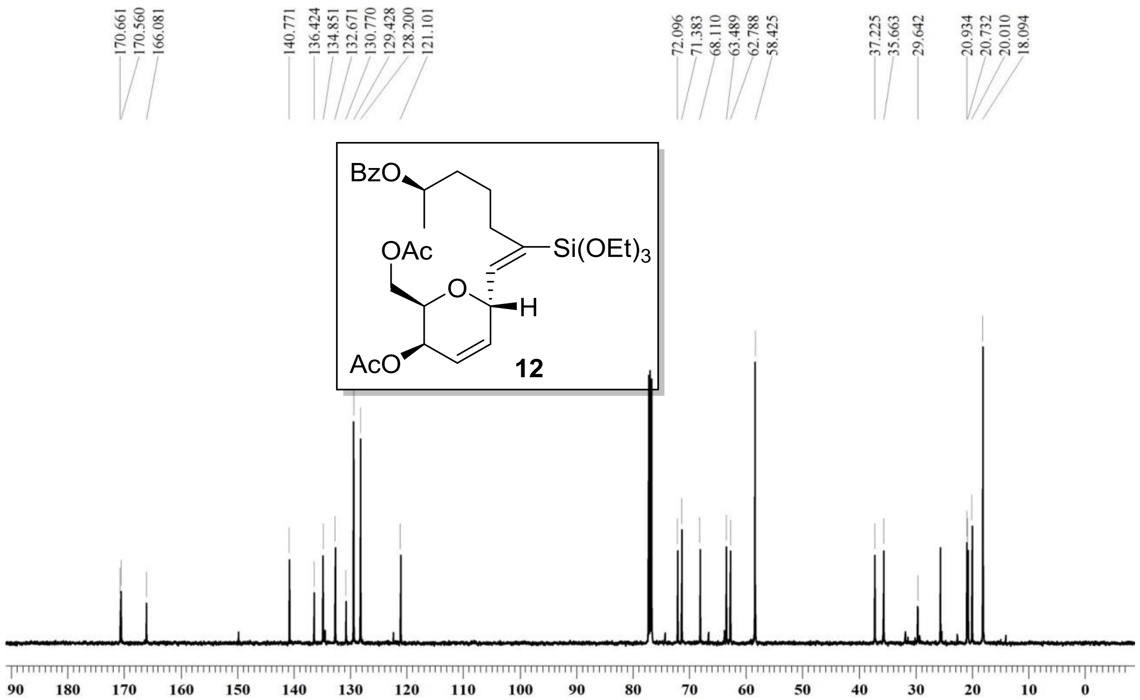

^{13}C NMR Spectrum of 12 (125 MHz, $CDCl_3$)

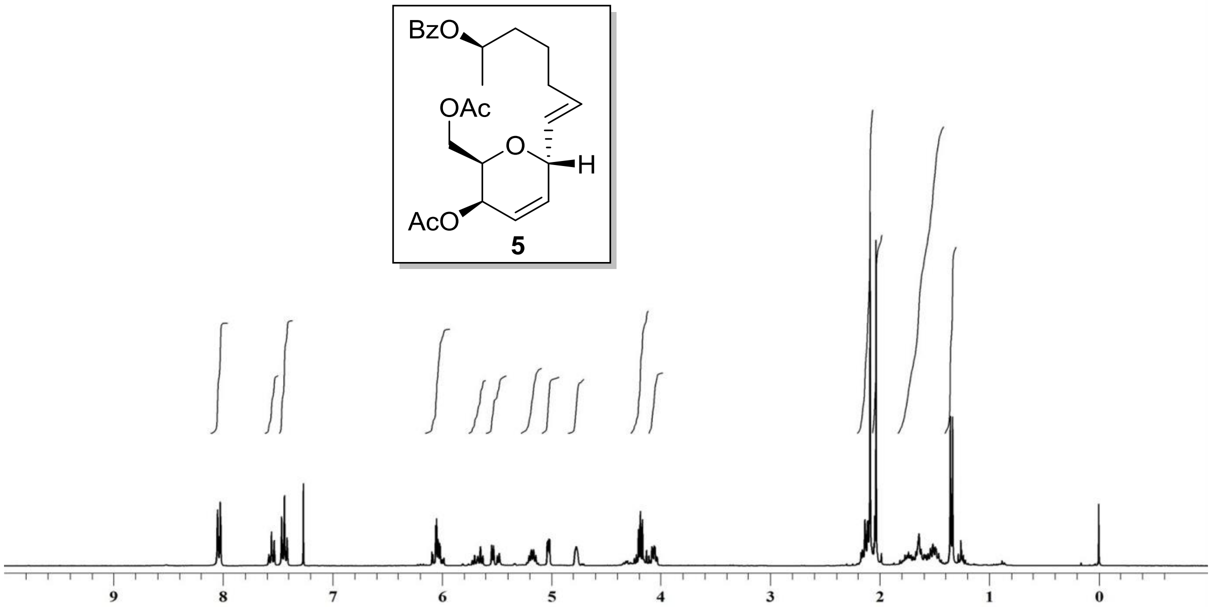

^{1}H NMR Spectrum of 5 (300 MHz, $CDCl_3$)

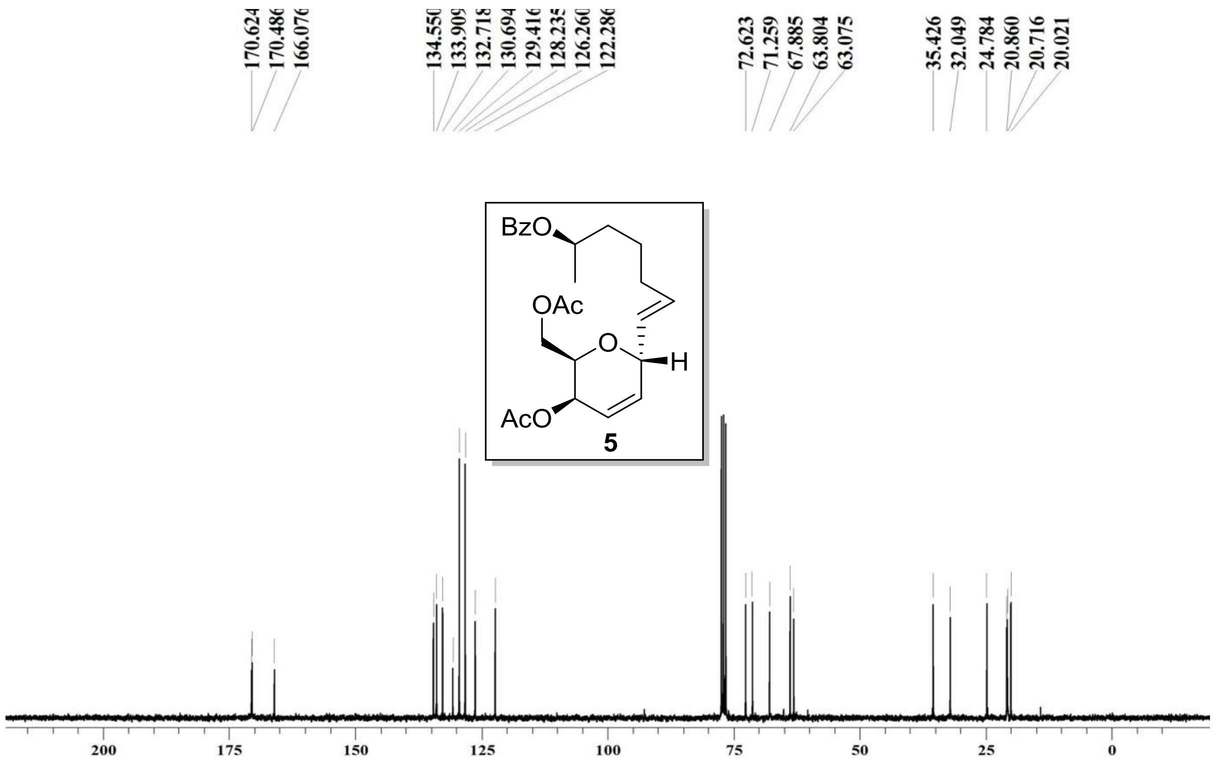

^{13}C NMR Spectrum of 5 (75 MHz, $CDCl_3$)

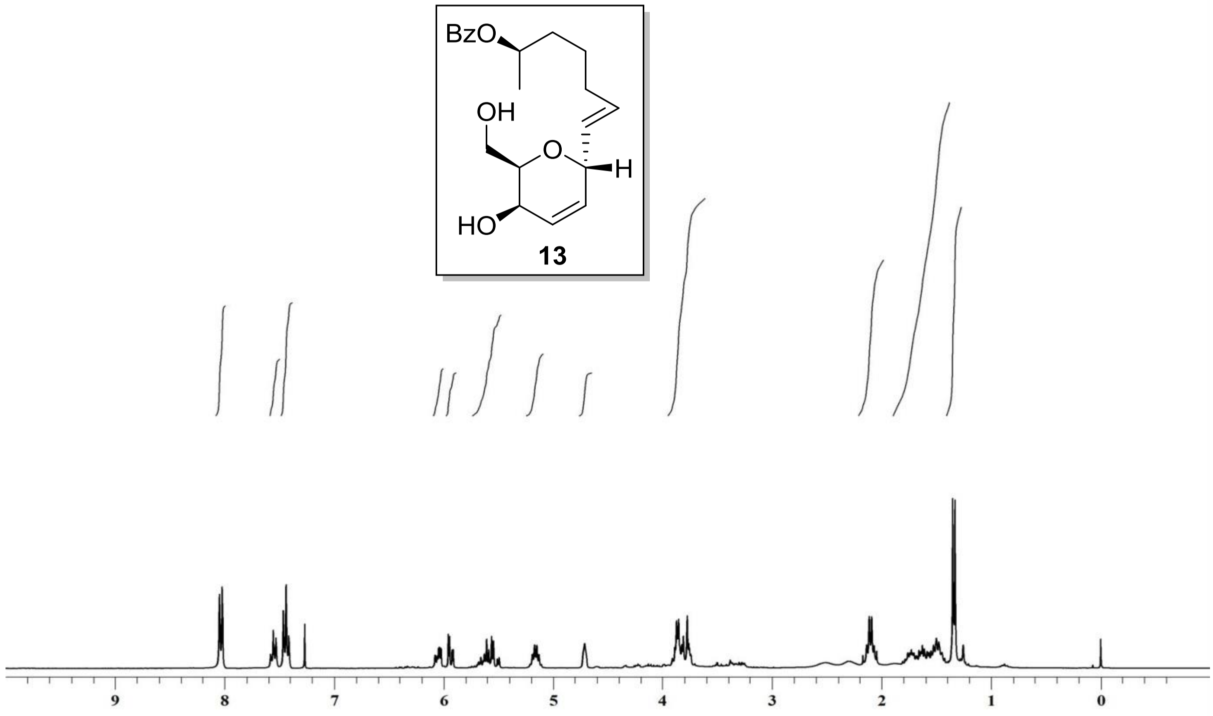

^{1}H NMR Spectrum of 13 (300 MHz, $CDCl_3$)

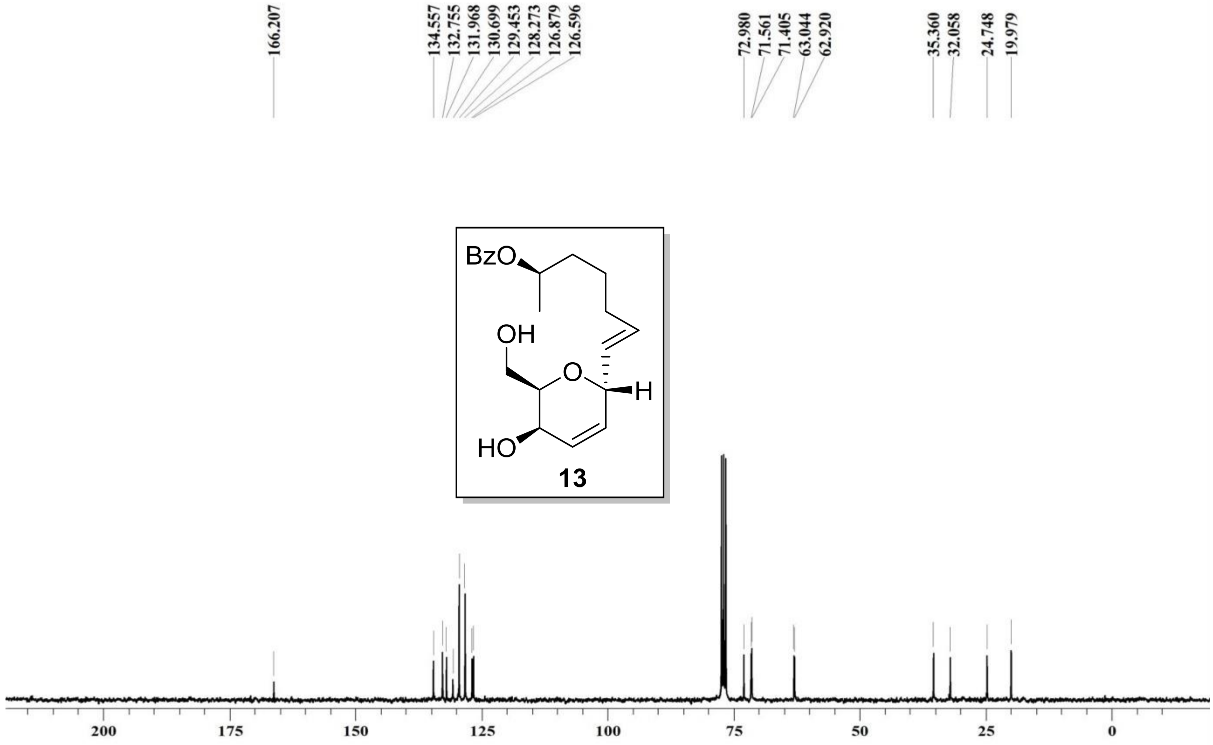

^{13}C NMR Spectrum of 13 (75 MHz, $CDCl_3$)

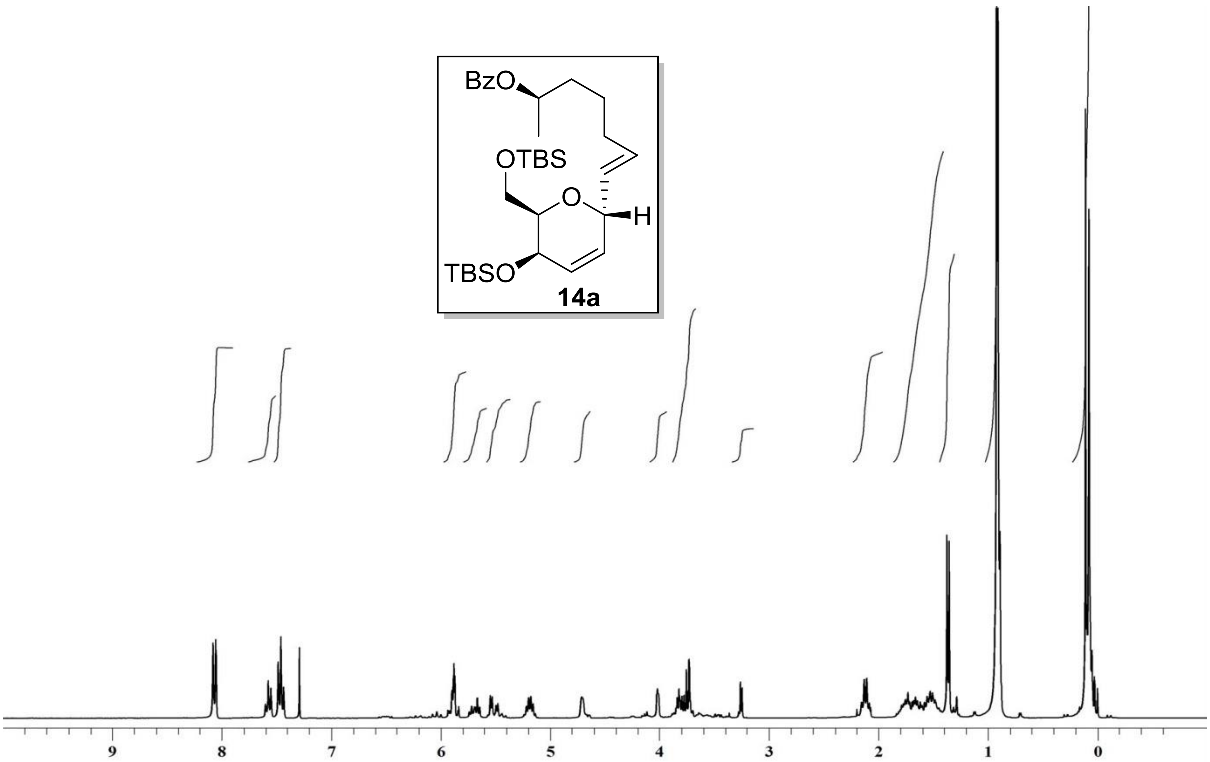

^{1}H NMR Spectrum of 14a (300 MHz, $CDCl_3$)

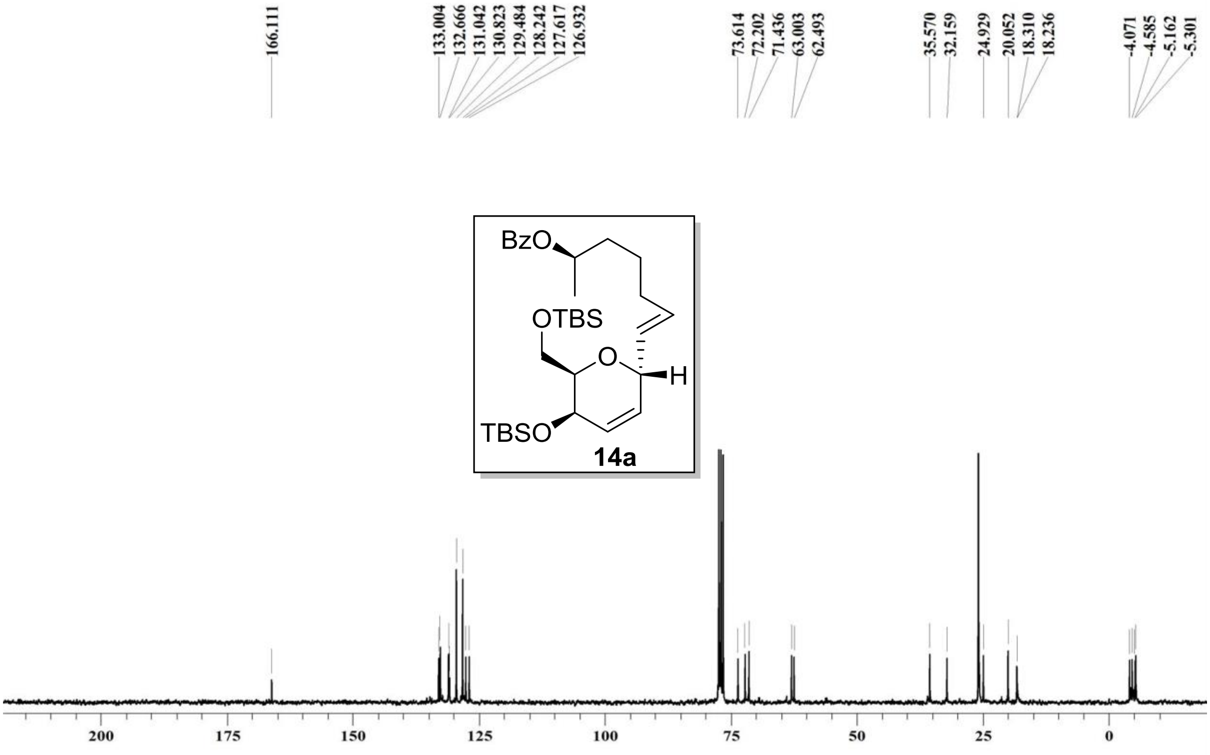

^{13}C NMR Spectrum of 14a (75 MHz, $CDCl_3$)

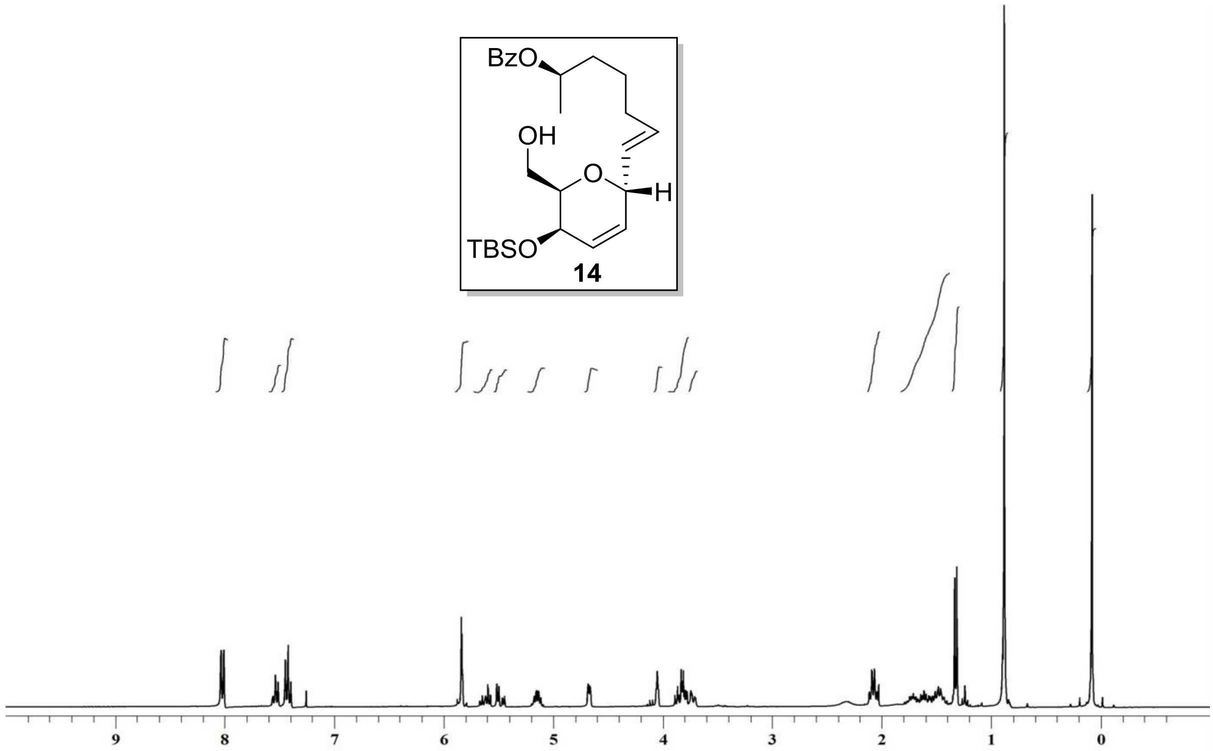

^{1}H NMR Spectrum of 14 (300 MHz, $CDCl_3$)

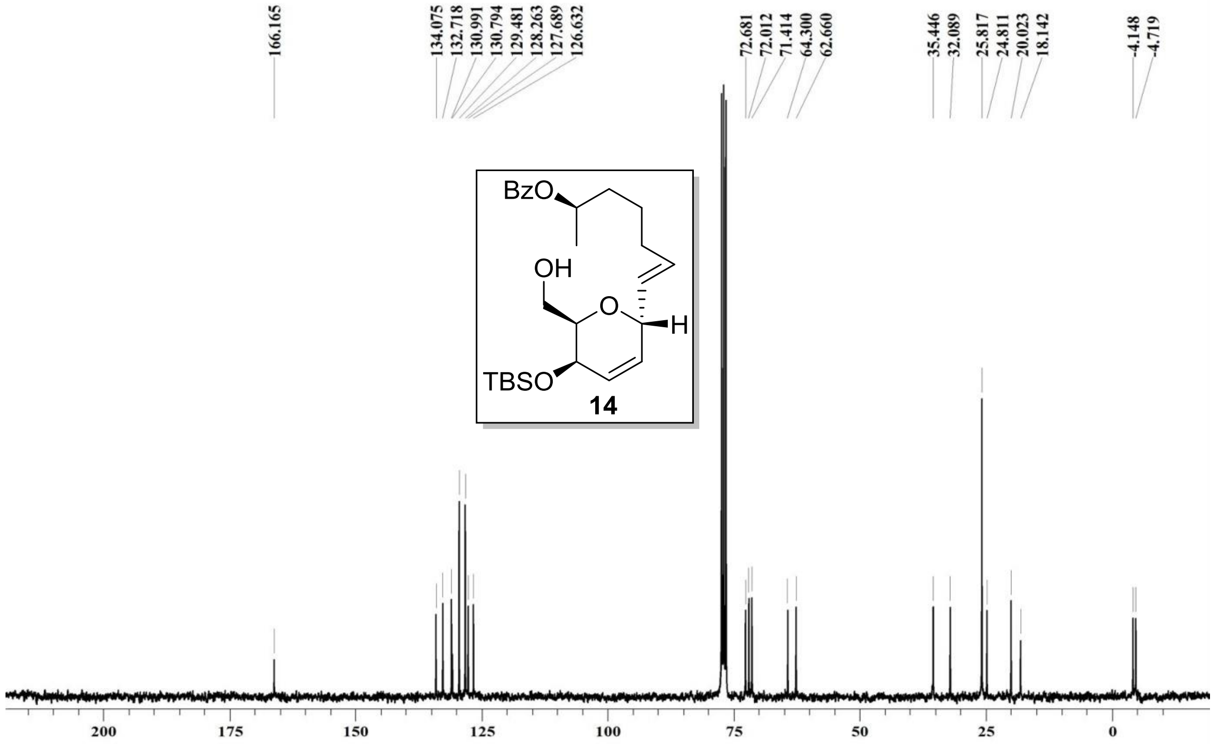

^{13}C NMR Spectrum of 14 (75 MHz, $CDCl_3$)

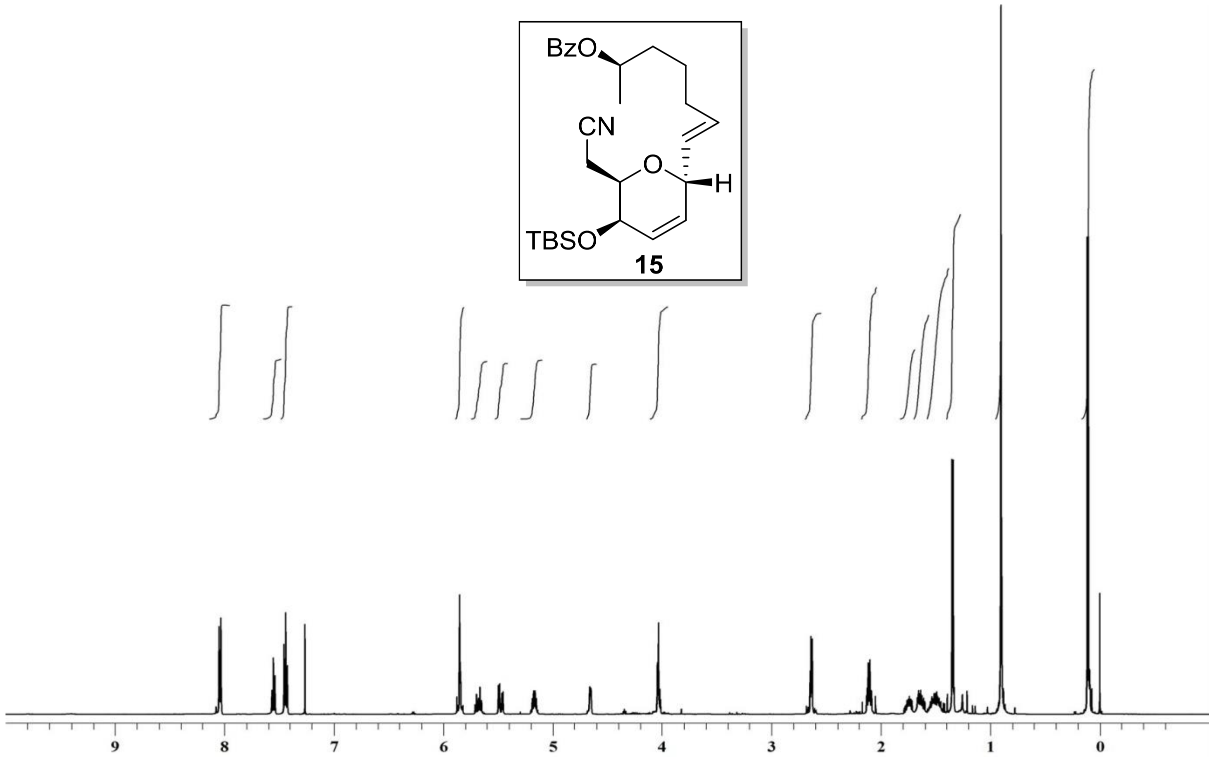

^{1}H NMR Spectrum of 15 (500 MHz, $CDCl_3$)

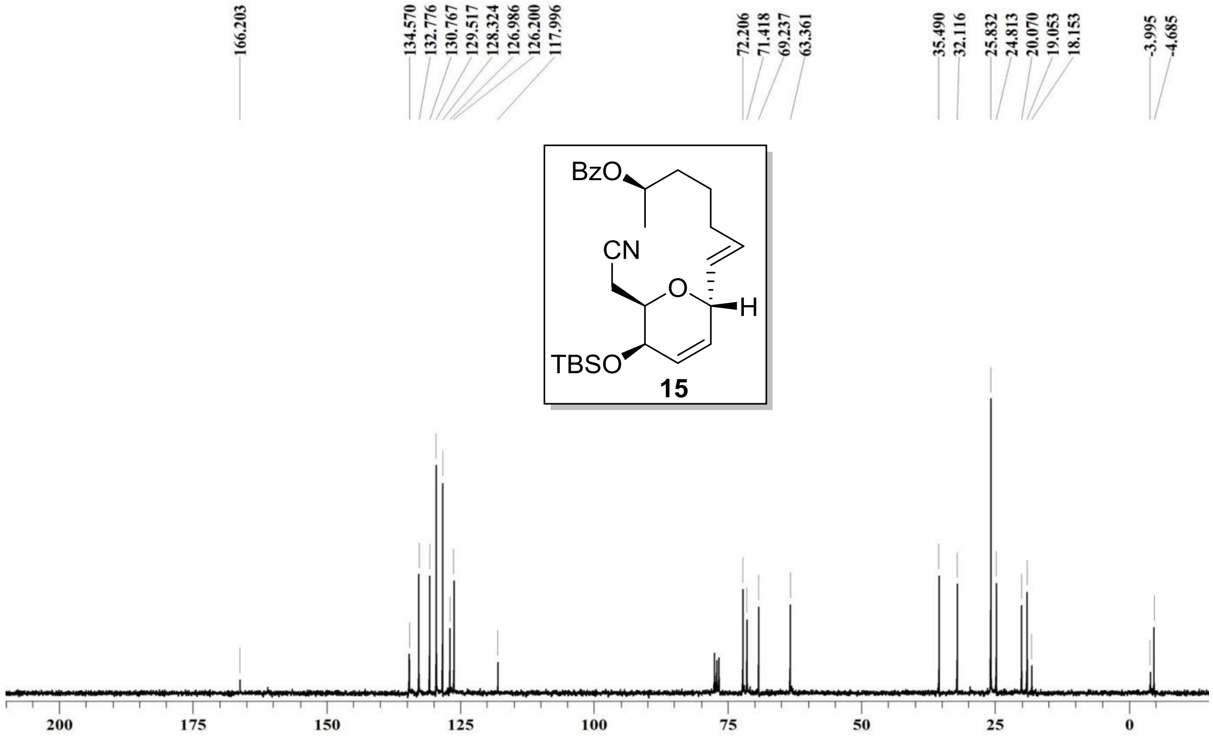

^{13}C NMR Spectrum of 15 (75 MHz, $CDCl_3$)

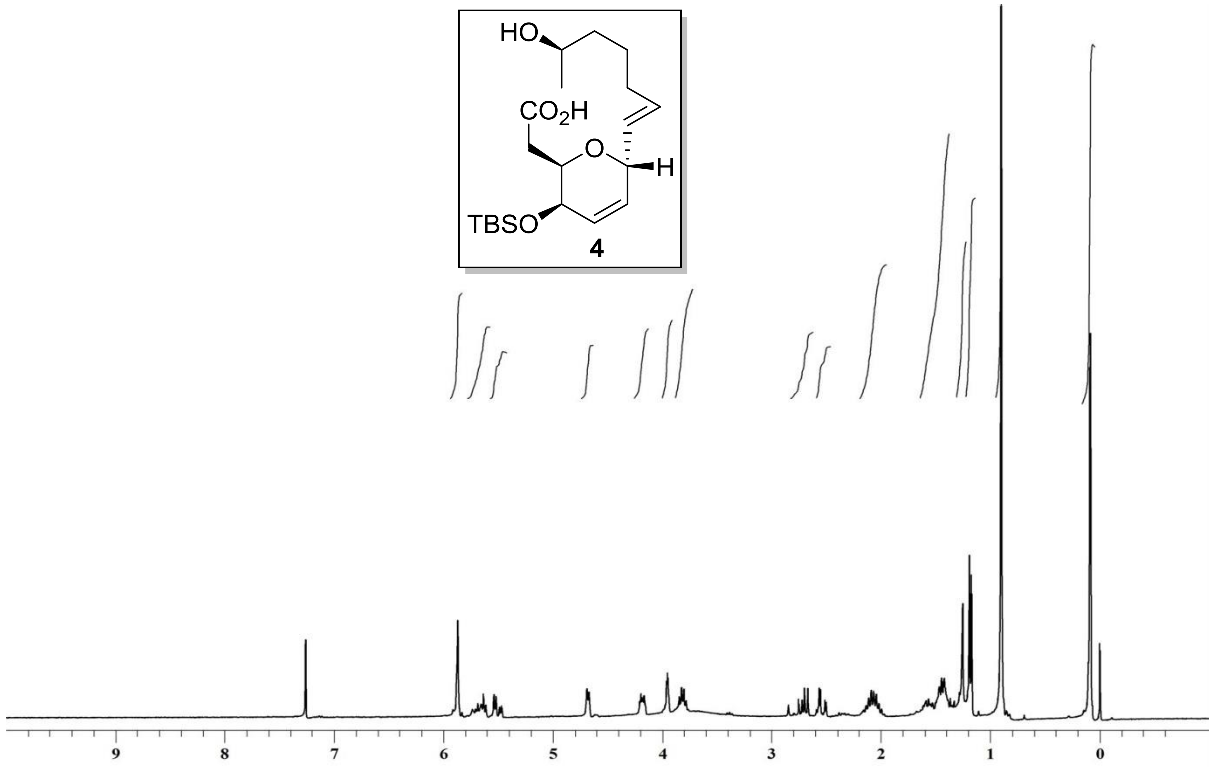

^{1}H NMR Spectrum of 4 (300 MHz, $CDCl_3$)

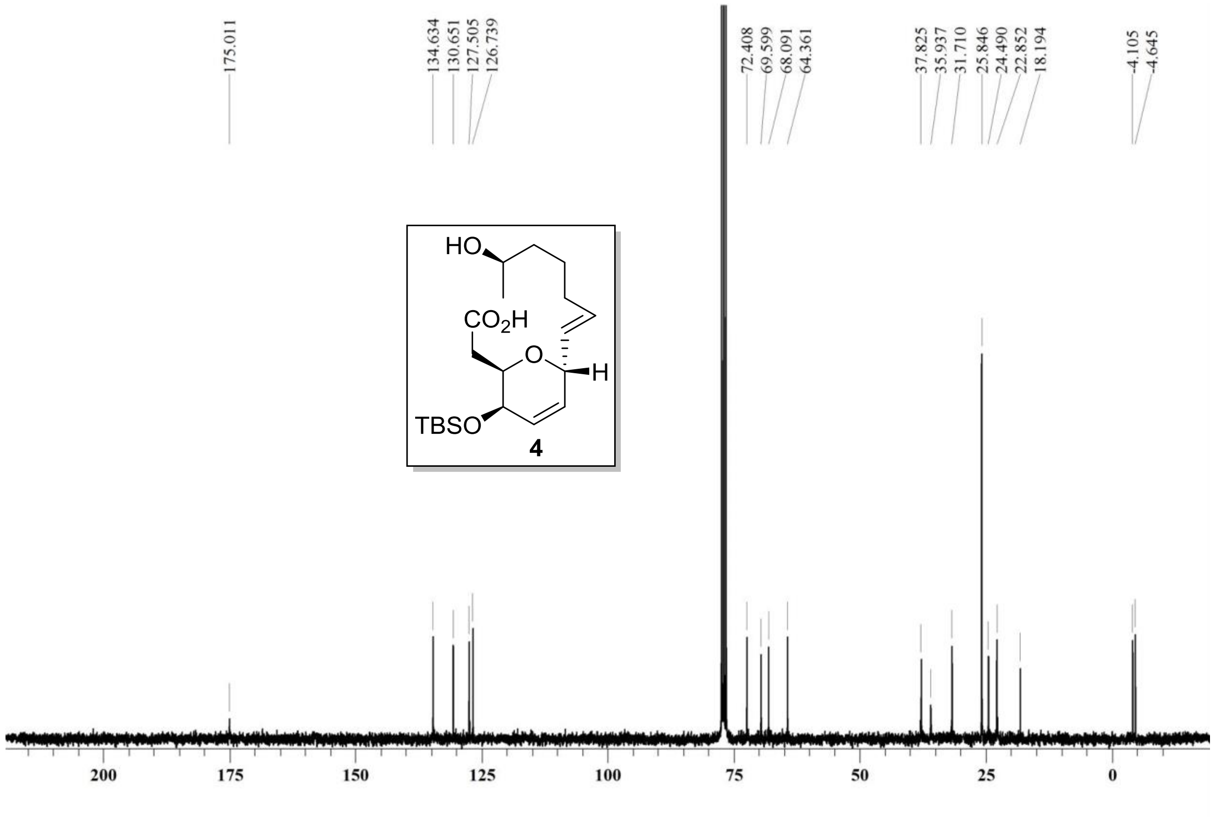

^{13}C NMR Spectrum of 4 (75 MHz, $CDCl_3$)
